# Insertion Depth Modulates Protein Kinase C-δ-C1b Domain Interactions with Membrane Cholesterol as Revealed by MD Simulations

**DOI:** 10.3390/ijms24054598

**Published:** 2023-02-27

**Authors:** Patrick T. Judge, Sarah A. Overall, Alexander B. Barnes

**Affiliations:** 1 Department of Biochemistry, Biophysics & Structural Biology, Washington University in St. Louis, St. Louis, MO 63130, USA; 2 Laboratory of Physical Chemistry, ETH Zürich, 8093 Zurich, Switzerland

**Keywords:** protein kinase C, cholesterol, bryostatin, phorbol esters, molecular dynamics, membrane bilayer, lipid rafts

## Abstract

Protein kinase C delta (PKC-δ) is an important signaling molecule in human cells that has both proapoptotic as well as antiapoptotic functions. These conflicting activities can be modulated by two classes of ligands, phorbol esters and bryostatins. Phorbol esters are known tumor promoters, while bryostatins have anti-cancer properties. This is despite both ligands binding to the C1b domain of PKC-δ (δC1b) with a similar affinity. The molecular mechanism behind this discrepancy in cellular effects remains unknown. Here, we have used molecular dynamics simulations to investigate the structure and intermolecular interactions of these ligands bound to δC1b with heterogeneous membranes. We observed clear interactions between the δC1b-phorbol complex and membrane cholesterol, primarily through the backbone amide of L250 and through the K256 side-chain amine. In contrast, the δC1b-bryostatin complex did not exhibit interactions with cholesterol. Topological maps of the membrane insertion depth of the δC1b-ligand complexes suggest that insertion depth can modulate δC1b interactions with cholesterol. The lack of cholesterol interactions suggests that bryostatin-bound δC1b may not readily translocate to cholesterol-rich domains within the plasma membrane, which could significantly alter the substrate specificity of PKC-δ compared to δC1b-phorbol complexes.

## 1. Introduction

Protein kinase C δ (PKC-δ) is a serine/threonine kinase that plays a central role in regulating cell proliferation [1], growth [2], apoptosis [3,4] and differentiation [5,6]. It is one of the novel PKC isoforms, along with ε, η and θ, which do not require Ca^2+^ for activation, unlike the conventional PKC isotypes, α, β and γ. PKC-δ is comprised of an N-terminal regulatory domain that is linked to a C-terminal catalytic domain by a flexible hinge region. The regulatory domain contains the C2-like domain, the pseudosubstrate domain, which auto inhibits the catalytic domain, followed by a membrane-binding C1 domain. The C1 domain is comprised of two zinc finger motifs, C1A and C1B, which bind agonists such as diacylglycerol (DAG) and phorbol esters. Upon agonist and membrane binding, PKC-δ undergoes a conformational change which releases the kinase domain from the pseudosubstrate and allows the binding and phosphorylation of substrates. Overexpression of PKC-δ is associated with increased apoptosis and cell cycle arrest in many cell types through p53 activation [2,7,8]. Conversely, the PKC-δ-mediated activation of NFκB is often associated with tumor progression in pancreatic [9], hepatic [10] and breast [11,12,13] cancers as well as with HIV latency reversal in T cells [14,15]. While these cell-type-specific effects complicate our understanding of PKC-δ signaling, the participation of PKC-δ in many signaling cascades gives rise to the attractiveness of PKC-δ modulators as drug targets for a wide range of applications, including HIV latency reversal [14,16,17], anti-cancer therapies [18,19,20,21] and Alzheimer’s treatments [22,23], many of which are currently in clinical trials [24,25].

The endogenous modulator of PKC-δ is diacylglycerol (DAG) which binds to the C1 domain [26,27]. The C1 domain comprises the C1a and C1b regulatory domains, of which the C1b domain (δC1b) is predominantly responsible for anchoring PKC-δ to the plasma membrane upon ligand binding [27,28,29], a process that requires the presence of phosphatidylserine (PS) [30,31]. Bryostatins and phorbol esters are two major classes of PKC-δ modulators that bind to the δC1b domain, but which have opposing effects on cells [16,32,33]. Both efficiently reactivate latent HIV provirus and bryostatins suppress inflammatory cytokine production, limiting T cell responsiveness [34,35,36], while phorbol esters promote excessive inflammatory cytokine production causing uncontrolled immune cell proliferation [37]. The mechanism behind these phenotypic disparities is not yet understood. Both molecules bind to δC1b with an affinity of K_d_ = 3–4 nM [38,39] and display a similar binding mode involving hydrogen bonding to the backbone amide of T242 and the backbone carbonyl of T242 and L251 [40,41] through an alkoxy group on the drugs. This is a binding mode recapitulated by DAG [41]. In addition, both classes of modulators also bind to other PKC isoforms [38,39]. Collectively, the study of ligand binding to PKC-δ has revealed that the structure and biochemistry of ligand interactions cannot fully explain the disparate cellular responses to different PKC-δ agonists.

The nature of the ligands’ interaction with membrane lipids could be a greater determinant of PKC-δ activity [42,43,44,45]. DAG acyl chain composition greatly affects the membrane recruitment of many PKC isoforms, with longer, more unsaturated chains providing the enhanced activation of novel and conventional PKCs [46,47]. The effects of acyl chain saturation on membrane order parameters are thought to be the driving force behind the phenomenon [44]. Decreased membrane order and the formation of non-lamellar phases are associated with increased PKC activity [48,49,50]. The cooperative effects of DAG and cholesterol on membrane order parameters further promotes PKC-δ recruitment to lipid bilayers and correlates with PKC-δ kinase activity [51,52]. The effect can be recapitulated with ceramides of increasing unsaturation instead of cholesterol, promoting non-bilayer phases, as determined by ^2^H solid-state NMR [49,53]. In addition, the nature of the ligand may influence the membrane-bound topology of δC1b in homogenous PS membranes [54]. Differences in the interaction of phorbol and bryostatin with water molecules appears to modulate the insertion depth of δC1b and was a clear distinction between the two modulators in molecular dynamics (MD) simulations, suggesting membrane insertion depth might somehow influence PKC-δ activity. 

The requirement for PS in PKC-δ activity has led to most MD simulations [44,54,55] and experimental studies [39,48,56] using PS or phosphatidylserine:phosphatidylcholine (PS:PC) model membranes to study PKC activity, which does not reflect the complex composition of the plasma membrane. Given the sensitivity of PKC-δ activity and membrane binding to lipid composition, it is clear the membrane environment beyond the presence of PS is important. However, lipid interactions with peripheral membrane proteins are difficult to detect experimentally without prior knowledge being used to guide the design of appropriate experiments. This is especially difficult in complex membrane systems. Here, we use all atom molecular dynamics simulations to explore the interactions between different δC1b-ligand complexes and heterogenous membranes mimicking the plasma membrane composition, specifically analyzing differences in how these complexes interact with cholesterol. We provide a rationale for the mechanism by which δC1b insertion depth might influence cell signaling as a guide for future experimental studies.

## 2. Results

### 2.1. δC1b-Phorbol and δC1b-Bryostatin Show Differential Lipid Headgroup Engagement

**Figure 1:**
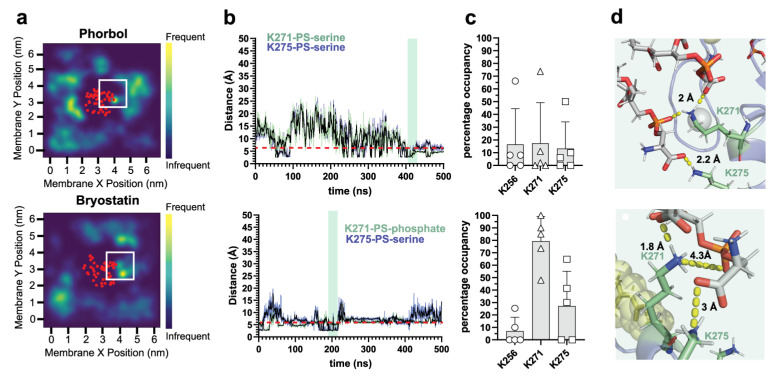
Interactions between PS and lysine side chains of δC1b. Top panels show δC1b-phorbol simulations. Bottom panels show δC1b-bryostatin simulations. (**a**) Heatmaps show the localization of PS lipids in the inner leaflet over a 500 ns simulation period and are representative of five simulations (see Figure S2 for full dataset). The red dots indicate the average position of the backbone Cα of δC1b. (**b**) Interaction lifetimes of PS-lysine interactions. The dotted line indicates the 6 Å cutoff used to determine occupancy in (**c**). The shaded area indicates the time frame in which the structures shown in (**d**) are obtained. (**c**) Total occupancy time of different lysine residues with PS over all five simulations. Bars indicate the mean, error bars represent the standard error of the mean and each point represents a single independent simulation. (**d**) Representative structural interactions from the shaded area in (**b**). PS lipid is colored grey sticks while δC1b-lysine residues are colored pale green sticks. Charge–charge interactions are indicated by the dashed yellow line and the interaction distance is indicated above the residue to which PS is interacting.

To assess the potential for δC1b-ligand complexes to interact with different membrane components, we simulated δC1b bound to phorbol and bryostatin (Figure S1) in heterogenous membranes mimicking the plasma membrane based on the composition used by [57] and comprising 25% cholesterol. Membrane systems were equilibrated after pre-insertion of the δC1b-ligand complex and thus report on interactions post the initial membrane-binding event. We expected to observe extensive interactions with PS through lysine residues, which have been observed in reported MD simulations of the C1b domains of PKC-α [55] and PKC-δ [54], as well as demonstrated experimentally in the structurally analogous C2 [58,59] and C1a domains [27,28,60]. While PS interactions were observed for both ligand complexes (Figure 1a), long-lived interactions were predominantly observed with the δC1b-bryostatin complex. Ionic interactions of ≤6 Å between the phosphate and carboxyl moieties of PS with lysine amine groups of δC1b generally lasted for 100–200 ns and were sampled multiple times during the simulations (Figure 1b,d), on average occupying K271 for 80% and K275 for 26% of the δC1b-bryostatin simulation time (Figure 1c). In contrast, lysine residues of δC1b-phorbol complexes were occupied with PS for only 15% of the simulation time and exhibited reduced interaction stability manifesting as increased distance fluctuations as a function of time (Figure 1b), a phenomenon also reported in simulations of PKC-α in PS:PC membranes [55]. Interestingly, δC1b contains five lysines, but K256, K271 and K275 accounted for almost all of the interactions observed with PS head groups, irrespective of the bound ligand. The close proximity of these lysines to one another also promoted multivalent interactions. In our simulations, multiple lysines were simultaneously engaged with the same PS molecule as well as with multiple PS molecules (Figure 1d), which has been suggested to contribute to the high affinity of PKC for PS membranes [55]. The simulation data is therefore consistent with our experimental knowledge of PKC-δ and its requirement for PS in membrane binding, but also revealed a significant disparity between phorbol- and bryostatin-bound complexes in the occupancy of lysine residues with PS. 

**Figure 2:**
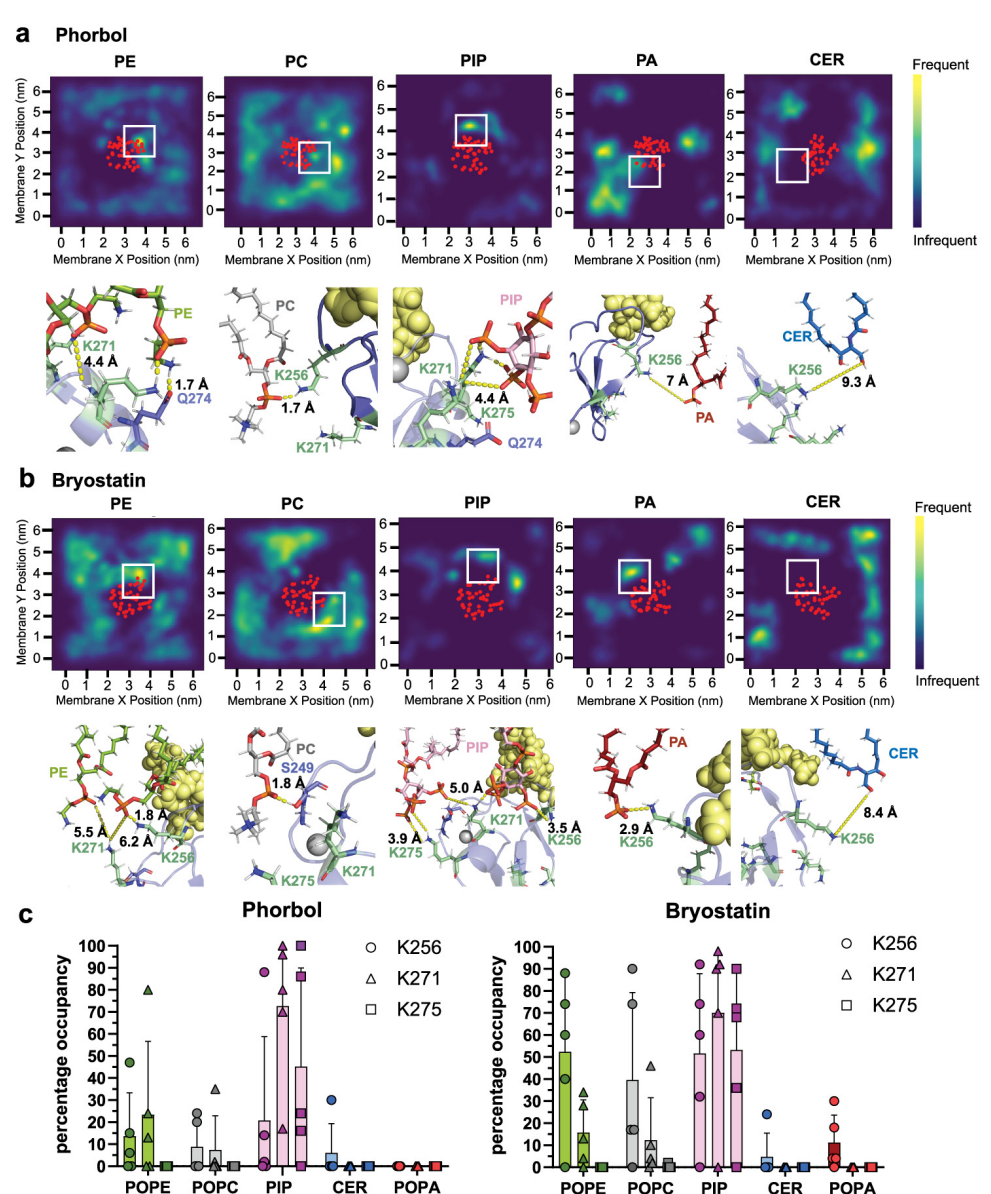
Interactions between δC1b and heterogenous lipids. Interactions between δC1b-phorbol (**a**) and δC1b-bryostatin (**b**) with phosphatidylethanolamine (PE), phosphatidylcholine (PC), phosphatidylinositol (PIP), phosphatidic acid (PA) and ceramide (CER). Heatmaps show the localization frequency of each lipid in the inner leaflet over 500 ns and are representative of five simulations. The red dots indicate the average position of the backbone Cα of δC1b. Structural interactions shown are representative of interactions in the region indicated by the white box. Interaction distances are indicated by dashed yellow lines, ligands are shown as yellow spheres and lysine residues are shown as pale green sticks. (**c**) Quantitation of the occupancy of K256 (circles), K271 (triangles) and K275 (squares) with various lipids across five simulations covering 500 ns each (2.5 μs in total). Bars indicate the mean, error bars represent the standard error of the mean and each point represents a single independent simulation.

To determine whether this could be explained by occupancy with other lipids we analyzed the interaction of δC1b-lysine residues with other types of lipids. In δC1b-phorbol simulations we observed transient interactions (<10 ns continuous interaction time or <50 ns total interaction time) with phosphate groups of most phospholipids but found the highest occupancy with phosphatidylethanolamine (PE), phosphatidylinositol (PIP) and, to a lesser extent, phosphatidylcholine (PC) (Figure 2a–c). In fact, interactions with PE and PIP exceeded those observed with PS. δC1b-bryostatin complexes also showed significant interactions with PE, PIP and PC. Short–lived interactions with phosphatidic acid (PA) were also observed. No short-range stable interactions were observed between ceramide and δC1b, suggesting ceramide does not interact with surface residues of ligand-δC1b complexes, consistent with the PKC-δ literature. Most of the interactions were concentrated around K256 with no preference for PS. δC1b interactions with PIP were particularly long-lived, forming stable, close (2.6–5 Å) interactions centered around lysines 256, 271 and 275. These interactions were particularly multivalent with other surrounding residues readily participating in the coordination of the multiple phosphates of PIP (Figure 2 and Figure S3). The distribution of δC1b occupancy with various lipids reflects the dynamic nature of the heterogenous membrane. The exchange of lipid groups with one another for residue occupancy is a significant contributor to this distribution. As well as factors such as PIP engagement, which was especially long-lived (Figure S3); thus, whether PIP became engaged early or late in the simulation greatly influenced the net interaction time with other lipids, as the sheer size and charge density of PIP effectively excluded other lipids from the interacting residue. This resulted in a large distribution of residue occupancies across simulations, which would likely even out over a longer simulation period. Together these data reveal δC1b to be stabilized by interactions with a range of lipids through its lysine residues and that membrane association after the initial binding step does not appear to be particularly stabilized by PS association. 

### 2.2. δC1b-Phorbol Interacts with Cholesterol in Heterogenous Membranes

**Figure 3:**
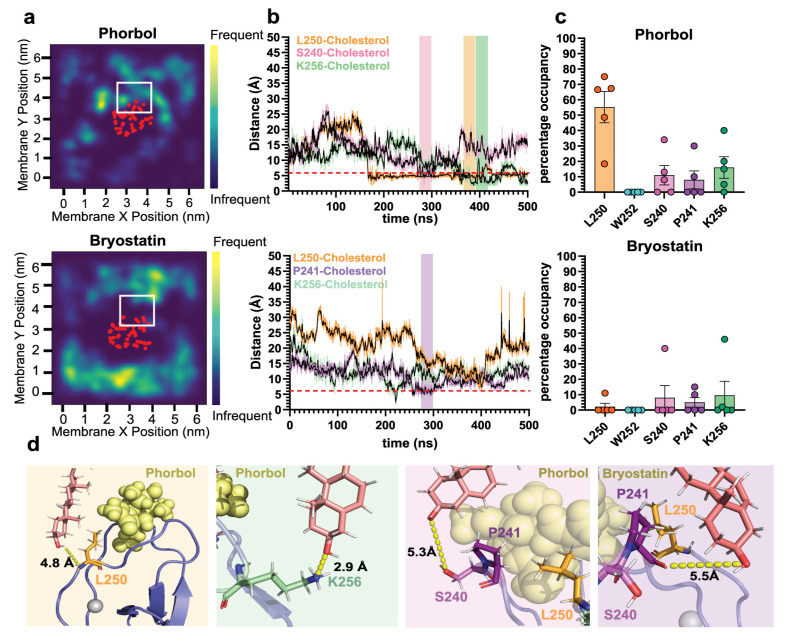
Interactions between cholesterol and δC1b. Top panels show δC1b-phorbol simulations. Bottom panels show δC1b-bryostatin stimulations. (**a**) Heatmaps show the localization frequency of cholesterol in the inner leaflet over 500 ns. The red dots indicate the average position of the backbone Cα of δC1b. Plots are representative of five simulations (full dataset is shown in Figure S4). (**b**) Interaction lifetime of L250 (orange), S240 (pink), K256 (green) and P241 (purple) with cholesterol. The dashed line indicates the 6 Å cutoff used to quantify residue occupancy in (**c**). (**c**) Bar graphs show the mean occupancy of residues with cholesterol across all simulations. Bars indicate the mean, error bars represent the standard error of the mean and each point represents a single independent simulation. (**d**) Representative structural interactions extracted from the simulation time frame indicated by the shaded regions in (**b**). Ligands are shown as yellow spheres, L250 is shown as orange sticks, S240 as pink sticks, P241 as purple sticks and K256 as pale-green sticks.

Cholesterol is known to enhanced PKC binding to model membranes, primarily through changes in bilayer order parameters [51,52]. Interactions between cholesterol and C1b have never been observed, although the ligand’s interaction with cholesterol remains unknown. To address this, we analyzed the interactions of δC1b-ligand complexes with membrane cholesterol. Heatmaps of cholesterol localization frequency showed the accumulation of cholesterol at the ligand binding loops of δC1b-phorbol complexes but not in δC1b-bryostatin simulations (Figure 3a). Examination of structures from the simulation revealed no significant direct interactions between cholesterol and phorbol. Instead revealing the accumulation of cholesterol around the binding loops to be due to hydrogen bonding between the amide proton of L250 and the cholesterol hydroxyl (Figure 3d). These interactions were long-lived, lasting for 100–400 ns and occupying L250 for an average 50% of the total simulation time (Figure 3b,c). Interaction lifetimes of δC1b-phorbol with cholesterol exceeded those observed with PS and were largely absent from δC1b-bryostatin simulations. More transient interactions were observed with S240 and K256, exhibiting an average occupancy of 11% and 16%, respectively. An occupancy time equivalent to that of PS interactions (Figure 1c). It can be observed from the interaction lifetime plots that δC1b-phorbol could be engaged with multiple cholesterols at multiple sites simultaneously (Figure 3b), raising the possibility of an avidity-enhancing δC1b-cholesterol association. 

**Figure 4:**
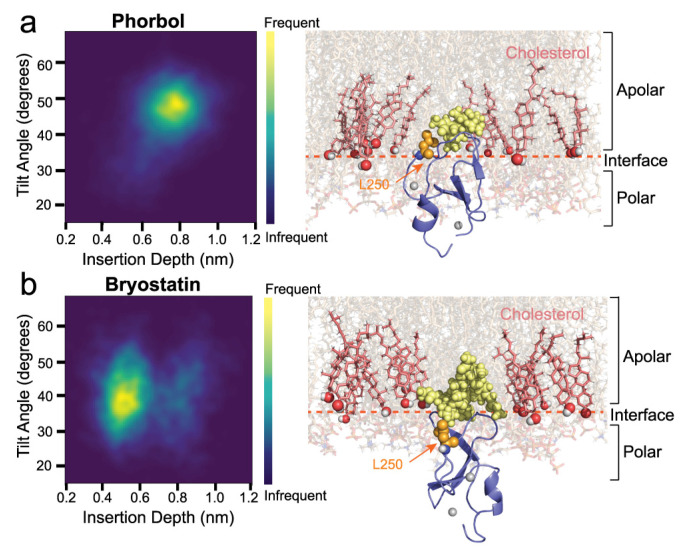
Topology of δC1b-ligand complexes. Topology of δC1b bound to (**a**) phorbol and (**b**) bryostatin. Each heatmap is an average for all five simulations for each complex over a total 2.5 μs simulation time. Areas of greater intensity represent topologies more frequently found in each simulation. Structural snapshots show the positioning of the L250 residue (orange spheres) relative to the cholesterol hydroxyl (shown as red spheres). The polar:apolar interface is indicated by the dashed red line and is positioned at the average position of the glycerol backbone of phospholipids.

Only one of five bryostatin simulations showed a significant interaction between S240 or K256 (Figure 3b,c), and to a lesser extent L250. Manual inspection of the simulations suggested L250 to not be accessible to cholesterol in most simulations due to a shallower membrane topology when bound to bryostatin. Topological heatmaps of the average insertion depth and tilt angle of δC1b-ligand complexes shows δC1b inserts deeper into the membrane when bound to phorbol (Figure 4a), averaging an 8 Å insertion depth and 48° tilt angle compared to 5 Å when bound to bryostatin and with a 38° tilt angle. This is consistent with previous studies [54]. Though the effect appears small, the change in topology positions L250 outside the membrane interface and it is no longer accessible to cholesterol for most of the simulation time (Figure 4b). The sustained interaction of S240 and P241 observed in one of five simulations was predicated on a deeper binding mode in this simulation (Figure S5). Regardless, it can be seen that δC1b-phorbol is significantly engaged in cholesterol interactions, particularly through L250, and this appears to be modulated by insertion depth and tilt angle. An increased tilt with respect to the membrane normal tilts the L250 up towards the membrane, increasing its membrane penetration depth and accessibility to cholesterol, even when the protein’s insertion depth becomes shallower. Thus, the shallower binding mode elicited by the bryostatin-bound complex and the upright positioning of the protein may have an important role in dictating the potential interactions δC1b has with surrounding lipids and its inclusion or exclusion from cholesterol-rich regions. Since the C1b domain is the primary determinant of membrane association of the novel class of PKCs [27,28,29], this could influence PKC-δ accessibility to particular substrates.

### 2.3. δC1b-Merle27 Mimics δC1b-Phorbol in Insertion Depth and Cholesterol Interactions

**Figure 5:**
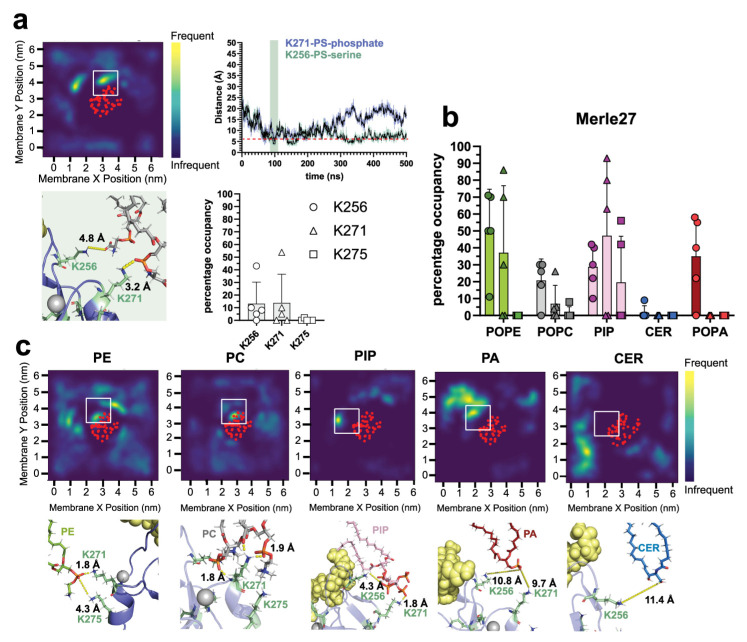
Lipid interactions with δC1b-Merle27. (**a**) Interactions between δC1b-Merle27 and PS. Representative heatmap indicates the frequency of PS in the lower leaflet over 500 ns (complete data set is shown in Figure S2). Interaction lifetimes are shown in the xy plot for the residues indicated. Example hydrogen bonding patterns are shown in the green box and are taken from the shaded region of the interaction lifetime plot. Quantification of PS-lysine interactions are shown in the grey bar graph. (**b**) Quantification of the interaction of δC1b-Merle27 with various lipids. (**c**) Heatmaps showing the frequency of lipid localization in the inner leaflet over the 500 ns simulation time. Heatmaps are representative of five simulations and the red dots indicate the average position of the Cα backbone of δC1b. Representative structures are shown below from regions indicated by the white box. Lysine residues are shown as pale-green sticks and the ligand is represented as yellow spheres. Bars indicate the mean, error bars represent the standard error of the mean and each point represents a single independent simulation.

To determine whether insertion depth can explain the distinct biological function of phorbol and bryostatin, we simulated a bryostatin analogue, Merle27. Merle27 is known to elicit cellular responses that are similar to those initiated by phorbol [61], despite its structural similarities to bryostatin (Figure S1). We observed interactions between PS and δC1b-Merle27 lysine residues (Figure 5a,b), predominantly occupying K256 and K271 for an average of 13% of the simulation time (Figure 5a), similar to that observed in δC1b-phorbol simulations (Figure 1c). We also observed increased interactions between δC1b-Merle27 and PE, PC, PIP and PA compared to PS, particularly through K256, similar to that observed in δC1b-bryostatin simulations (Figure 2c and Figure 5c). There were also no interactions with ceramide, as observed for the phorbol and bryostatin complexes. 

**Figure 6:**
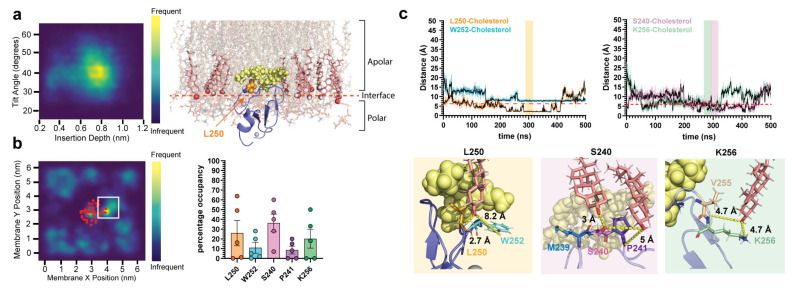
Topology of δC1b-Merle27 and its interaction with cholesterol. (**a**) Topology heatmap of δC1b-Merle27 showing the average insertion depth and tilt angle (averaged from five independent 500 ns simulations over a total of 2.5 μs). Representative snapshot showing the relative position of δC1b-Merle27 to cholesterol hydroxyl groups (red spheres) and the membrane interface. L250 is shown as orange spheres. The polar:apolar interface is indicated by the dashed red line. (**b**) Heatmap showing the localization of cholesterol in the inner leaflet across the 500 ns simulation time. Data shown are representative of five simulations (full dataset is shown in Figure S4). Residue occupancy with cholesterol is quantified in the bar graph across all five simulations. Bars indicate the mean with the standard error of the mean shown as the error bar and each point represents a single independent simulation. (**c**) The interaction lifetime between W252 (cyan), L250 (orange), S240 (pink) and K256 (green) with cholesterol-hydroxyls is plotted. Representative structures from the region indicated by the shaded area in the interaction lifetime plot are shown with the key residues indicated. Cholesterol molecules are shown as pale red sticks.

A comparison of topological heatmaps of the δC1b-ligand complexes in Figure 3 with those of δC1b-Merle27 (Figure 6a) shows the δC1b-Merle27 complex to insert deeper into the membrane, at 6.2 Å, but with a tilt angle in between bryostatin and phorbol at 43°. The reduced tilt angle in comparison to phorbol simulations may influence the participation of K256 in interactions with lipid headgroups as this does influence the membrane positioning of K256 (Figure S6). In addition, the δC1b-Merle27 complex exhibited a propensity to interact with cholesterol. We observed shorter-lived interactions between L250 and cholesterol compared to the δC1b-phorbol complexes (Figure 6b), but increased interactions with W252, S240 and K256, which persisted for >100 ns.

The C20 fatty acid of Merle27 extends out near L250, sterically hindering access of cholesterol to this residue and explaining the reduced interaction time observed (Figure S7). In δC1b-Merle27 complexes, the W252 side chain extended away from the ligand and showed a restricted ring-flipping motion compared to δC1b-phorbol complexes, which appears to be due to steric hindrance by the C21 methyl ester of Merle27 (Figures S7 and S8). This tryptophan has been shown to be important in the membrane binding of C1b domains by NMR, but only in the context of DAG binding and not phorbol ester binding [62,63]. In some instances, W252-cholesterol interactions were stabilized by additional hydrogen bonding with the backbone amide nitrogen of G253. δC1b-phorbol did not show interactions with W252, likely due to an increased side-chain motion, which we suggest inhibits long-lasting interactions with cholesterol (Figure S8). In δC1b-bryostatin simulations, it can be seen that the indole nitrogen of W252 is turned inwards and is hydrogen-bonded with bryostatin, stabilizing the protein–ligand complex (Figure S8). This makes the W252 side chain unavailable for interactions with cholesterol in the presence of bryostatin. We also observed the clustering of cholesterol molecules around the S240 and K256 side chain and backbone amide moieties (Figure 6c), engaging multiple cholesterols simultaneously. Overall, our simulations suggest a strong association between δC1b-Merle27 and cholesterol, albeit not site-specific and largely driven by ionic interactions with δC1b backbone amides and cholesterol hydroxyls. 

## 3. Discussion

The mechanism by which different PKC modulators cause disparate cellular phenotypes is unknown. Biochemical and structural studies find little difference in the interaction of modulators, such as phorbol and bryostatin, in terms of binding modes and affinity for PKC to explain the dichotomous phenotypes induced by these modulators. The role of ligand–membrane interactions is known to be important, whereby the ligand influences the insertion depth of PKC and effects membrane order, potentially modulating PKC accessibility to ligands within the membrane. Here, we provide the first indication that insertion depth modulation and ligand hydrogen bonding influences cholesterol interactions with PKC itself and hypothesize how this might impact cell signaling.

MD simulations are a powerful predictive tool for studying membrane–protein interactions [64,65]. Typically, simulations of PKC are conducted in homogenous PS membranes or PS:PC membranes, a composition also typically adopted experimentally. However, cellular membranes are much more complex compositionally. Despite it being difficult or impossible to capture the full complexity in simulations, studying a composition more reminiscent of cellular membranes can reveal previously unknown interactions that may be physiologically relevant. However, the prediction of potential interactions is required in order to design appropriate experimental approaches for detecting these associations. In heterogenous membranes, we observed interactions between the PS and lysine residues of δC1b consistent with published studies [54,55,60]. Stable interactions with other lipids, particularly PIP and PE but to a lesser extent PC and PA, were also identified and support experimental data demonstrating some degree of C1b affinity for anionic lipids and headgroup moieties [66]. However, the occupancy time depended on the bound ligand, with bryostatin promoting the most diverse and longest-lived interactions. The low occupancy of δC1b-phorbol is, however, consistent with published studies of PKCα-C1b [55] and may be reflective of the deeper membrane topology observed in phorbol complexes. This could place lysine residues closer to the interfacial region of the bilayer and beyond close interactions with headgroup moieties. This is supported by the reduced headgroup interactions observed in δC1b-Merle27 simulations, which also exhibited a deep insertion topology. Overall, we found little evidence of specificity for PS over other lipids. Our study does not report on the initial binding of δC1b to heterogenous membranes as the protein–ligand complex is pre-inserted into the bilayer before equilibration and subsequent simulation. The specificity of δC1b for PS membranes may be a requirement for the initial binding event but does not appear to specifically stabilize the complex after insertion beyond the provision of charge contacts which can be adequately supplied by other charged lipids [67]. Multivalent interactions between PS and δC1b-lysine residues could provide an order of magnitude increase in membrane-binding avidity and apparent affinity for PS, as suggested by Voth and colleagues [55]. We also observed multivalent interactions with PS, but especially PIP, with multiple lysine residues coordinating to PS and PIP molecules. While increased binding avidity undoubtedly contributes to the stability of the initial binding event, we believe that this only partly explains the experimental data. Mutagenesis studies have shown little effect of the mutation K271G on δC1b binding to PS membranes [68], a finding recapitulated in conventional PKCs [60]. Our observation of the key involvement of K271 in multivalent interactions (essentially bridging between K275 and K256) would suggest that this multivalent interaction is not essential or is at least redundant with other interactions. It is possible that the mutation of K271G alters the membrane-binding topology, allowing K260 and K234 increased participation in headgroup interactions during the initiation of membrane binding and possibly compensating for the loss of this K271 bridge.

The implication of membrane insertion depth as a distinction between δC1b-phorbol and δC1b-bryostatin were identified in MD simulations by Pande and co-workers in PS membranes [54]. Chelation of water molecules to bryostatin was suggested as a mechanism by which δC1b-bryostatin adopts a shallower binding mode in PS membranes, a topology we recapitulate in heterogenous membranes, suggesting the membrane composition does not determine δC1b insertion depth. However, we find cholesterol has a strong propensity to associate with more deeply embedded δC1b-ligand complexes which could be physiologically significant. Insertion depth appeared to modulate cholesterol interactions through the residues L250, S240 and K256. These interactions were largely absent from δC1b-bryostatin simulations. Furthermore, many of these interactions persisted for 100–200 ns within 6 Å suggesting stable associations [69]. There are no studies published to date (to our knowledge) on the potential for cholesterol to interact directly with PKC-δ. Conventional PKC kinase activity has been associated with cholesterol in terms of substrate specificity [70], which was due to a reduction in membrane order and the promotion of non-bilayer lipid phases [51,52,71]. Mutation studies of PKC-δ show that L250G mutants have significantly reduced kinase activity and membrane binding to PS bilayers [68]. Although this study does not report on the potential localization differences of the L250G mutant in bilayers containing cholesterol, the result most likely suggests a change in hydrophobicity by the L250G mutant. This may affect membrane insertion, as suggested by recent elegant work by Igumenova and colleagues [41]. Alternatively, the L250G mutant could exhibit increased flexibility about the backbone position of this residue due to the mutation to glycine which may influence stable ligand binding and may not reflect membrane association per se. Such an effect might also be predicted to influence long-lived interactions with cholesterol.

**Figure 7:**
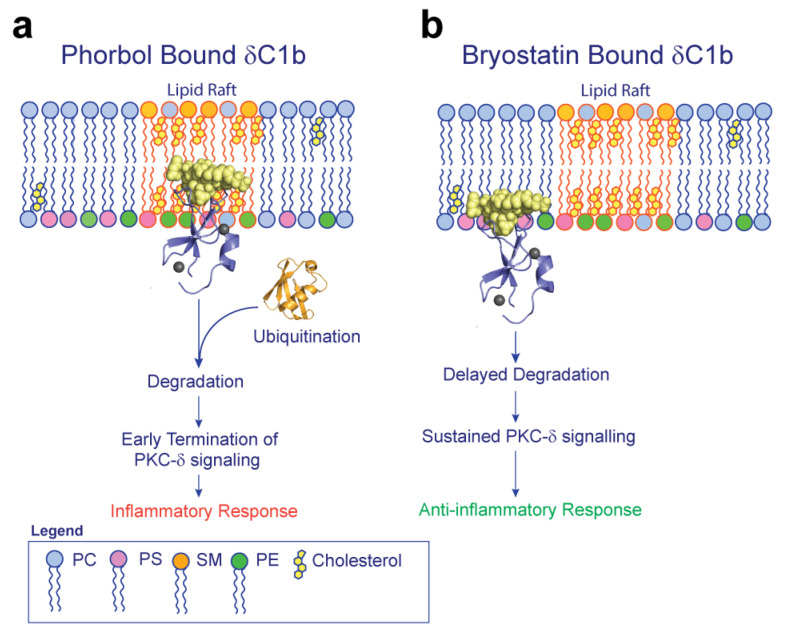
Schematic representation of the lipid raft hypothesis of PKC-δ modulation by phorbol and bryostatin. (**a**) Binding of phorbol results in deeper insertion within the membrane and contacts between PKC-δ and cholesterol are established. These contacts result in translocation of PKC-δ to cholesterol-rich lipid rafts, where ubiquitination occurs and rapid proteasomal degradation of PKC-δ ensues after initial activation [74]. (**b**) Binding of phorbol on the other hand results in a shallower binding topology and precludes the interaction between PKC-δ and cholesterol, limiting the translocation of PKC-δ to lipid rafts. Disparate localization of PKC-δ from E3 ligases results in prolonged PKC-δ signaling and therefore an alteration to the downstream transcriptional profile.

In model membrane systems, the addition of cholesterol increases the propensity of PS bilayers to form non-lamellar phases [52]. PS promotes non-bilayer phases and lowers bilayer order due to its small headgroup to tail volume ratio [72]. This phenomenon is enhanced by the presence of unsaturated acyl chains, particularly associated with DAG. The recruitment of PKC to cell membranes is increased in the presence of polyunsaturated DAG, the endogenous ligand of the PKC C1 domain, and is linked to enhanced PKC activity [43]. Recent MD simulations provide a rationale for this effect, suggesting DAG unsaturation increases solvent accessibility of the glycerol alkoxy, presumably increasing its availability for PKC binding. The effect was enhanced by the presence of cholesterol [73], essentially suggesting a shallower binding mode of DAG with increasing acyl chain unsaturation correlated with increased PKC activation [47,48]. This shallower binding topology may indeed increase PKC accessibility to membrane-embedded ligands. It is currently unclear whether this shallower binding mode is maintained after δC1b engagement, but it is likely that that the thermodynamic landscape shifts after PKC binding due to hydrogen bonding of the DAG alkoxy group by C1b, promoting deeper insertion of the hydrophobic surface formed by the ligand [41]. As we observed significant differences in membrane insertion depth of δC1b when bound to different ligands and within identical membrane compositions, we do not think that headgroup composition influences the membrane insertion depth of δC1b after the initial binding event. In addition, the calculated insertion depths are equivalent to that observed in PS homogenous membranes [54]. Therefore, it seems likely that the ligand alone determines the δC1b insertion depth. Thus, we hypothesize that after the initial binding event, the ligand’s interaction with water influences the insertion depth of the protein which determines cholesterol interactions and the potential to associate with cholesterol-rich regions, driving lipid raft partitioning (Figure 7). The interactions we observed are not cholesterol binding sites per se, but we suggest that the backbone orientation of residues in the β12 and β34 loops expose backbone amides for cholesterol–hydroxyl interactions and are thermodynamically favorable when δC1b inserts deeply into the hydrophobic environment of the inner bilayer. The potential for cholesterol to directly interact with the L250 backbone amide of δC1b bound to phorbol could be experimentally determined through NMR studies using isotopically labeled δC1b and ^13^C-labeled cholesterol. As the interaction appears to be somewhat dynamic, we would expect low dynamic systems and methods such as low temperature solid-state NMR to perform the best at demonstrating this potential interaction.

Lipid raft partitioning has long been associated with the spatial regulation of cell signaling [75,76]. It is known that phorbol esters promote the partitioning of PKC-δ into lipid rafts [77], followed by its rapid degradation [74]. While the microdomain localization of PKC-δ following bryostatin stimulation is unknown, the degradation of PKC-δ is delayed following bryostatin stimulation, which has tangible outcomes on the differentiation of keratinocytes [78]. As the cellular phenotypes of phorbol-stimulated cells compared to bryostatin-stimulated cells recapitulates the phenotypes of PKC-δ deficient [79,80] and PKC-δ overexpressing cells [7,81,82], respectively, we hypothesize that the temporal regulation of PKC-δ degradation could adequately explain the phenotypic differences observed between these two drugs. The hypothesis is readily testable by observing the colocalization of bryostatin-bound PKC to non-raft microdomains within the plasma membrane, either by Western blotting of fractionated cell lysates or super resolution microscopy. The spatiotemporal regulation of cell signaling is not new and has been suggested as a mechanism for PKC regulation by Newton and colleagues [83,84]. Elegant work by Berg and coworkers also demonstrate the sensitivity of T cell phenotypes to this kind of regulation [85]. The temporal delay of transcription factor translocation into the nucleus by weak TCR agonists is sufficient to attenuate the T cell response, despite the identity of involved transcription factors between strong agonists and weak agonists being the same. We hypothesize that PKC-δ could be subject to this kind of regulation and that differential raft-mediated signaling is one mechanism by which these pathways could be regulated. 

## 4. Materials and Methods

### 4.1. Preparation of Protein–Ligand Complexes

#### 4.1.1. δC1b-Phorbol Complex

The crystal structure of the mouse δC1b regulatory domain of PKC-δ bound to phorbol 13-acetate was obtained from the RCSB Protein Data Bank (PDB ID: 1PTR). The Orientation of Proteins in Membranes (OPM) PPM server [86] was used to obtain a structure with the initial orientation and insertion depth into a membrane and was saved as a PDB file. This structure was then used in the Membrane Builder module of the online CHARMM-GUI interface [86,87], which creates a pre-equilibrated membrane around the inserted protein. A heterogeneous membrane bilayer with cholesterol, phosphatidylethanolamine (PE), phosphatidylcholine (PC), phosphatidylserine (PS), phosphatidic acid (PA), phosphatidylinositol (PIP) and ceramide (CER) was constructed with the lipid composition shown in Table 1. Lower leaflet numbers are slightly lower due to the presence of the inserted δC1b-ligand complex. A water height of 17 was used with the TIP3P water model, and a neutralizing amount of ions were added using the replacement method. The protein–ligand complex was parameterized with CGenFF and input files for GROMACS [88] were generated for use with the CHARMM36m [89] forcefield, with a system temperature of 310.15 K. This process was repeated for a total of 5 δC1b-phorbol systems.

#### 4.1.2. δC1b-Bryostatin and δC1b-Merle 27 Complexes

As above, the crystal structure of the C1b regulatory domain of PKC-δ bound to phorbol 13-acetate was obtained (PDB ID: 1PTR). The ligand was removed using the BIOVIA Discovery Studio Visualizer (BIOVIA (2020), Dassault Systèmes, San Diego, CA, USA). The bryostatin and Merle27 structures were sketched as mol2 files in the BIOVIA Discovery Studio Visualizer. The AutoDock Tools package [90] was used to create pdbqt files of the protein and ligands. The size of the three-dimensional grid box for docking was set to 18, 26, 16 (x, y, z) and centered at 10.707, 21.52, 25.199 (x, y, z). The docking was performed using an AutoDock Vina in Ubuntu 16.4 with exhaustiveness = 10 and energyrange = 5 kcal/mol. From the resulting files, one structure exhibited the expected interaction between the C1 carbonyl on bryostatin/Merle27 with Gly253. This structure was used to generate input files for GROMACS, with a membrane composition shown in Table 2. As with phorbol, this process was repeated for a total of 5 C1b-bryostatin and 5 C1b-Merle27 systems. The RMSD between the post-equilibration structures for the C1b-bryostatin and C1b-phorbol simulations was 0.398 Å, and 0.364 Å for the C1b-Merle27 and C1b-phorbol structures. RMSD fluctuations across the production runs as a function of time are shown in Figure S9b).

### 4.2. Molecular Dynamics Simulations

All 15 systems comprising the three δC1b/ligand complexes were energy minimized, equilibrated, and simulated using the CHARMM36m forcefield [91] with GROMACS. All systems were simulated out to 500 ns (2.5 μs total for each complex). Residue Y6 of the C1b-domain crystal structure was mutated to H6 to model human PKC. Cysteines involved in zinc ion coordination were deprotonated, and all other residues were protonated as dictated by their pKa values at pH 7.4. Zinc ions were restrained so that they remained coordinated within their zinc finger motifs with harmonic springs. A lack of restraints resulted in a loss of coordination of the zinc ions with their respective residues. Energy minimization was completed with the steepest descent method using a Berendsen thermostat and a barostat for a maximum of 5000 steps or until F_max_ < 1000 kJ/mol/nm was reached (Figure S9a). Equilibration for a total time of 1125 picoseconds with a pressure of 1 bar and reference temperature of 310.15 K was carried out. Equilibration was assessed based on the area per lipid stability (Figure S9d). All bonds with rigid hydrogen atoms were kept with the LINCS algorithm, and long-range electrostatic interactions were investigated with the particle-mesh Ewald algorithm. The production runs were carried out at 310.15 K with times steps of 2 fs using a Nose-Hoover thermostat and Parrinello–Rahman barostat. Periodic boundary conditions in x, y, z dimensions were used with a final box size of approximately 60 × 60 × 90 Å in x, y, z, respectively.

### 4.3. Data Analysis

Data analysis was carried out using home-written PYTHON code, including MDTraj [92] to read the trajectories into the code. Heatmaps were obtained by tracking the x and y position of the atoms listed below in the lower leaflet housing the protein–ligand complexes. Cholesterol heatmaps were generated by tracking the position the oxygen of cholesterol and the amide nitrogen of L250, indole nitrogen of W252, side-chain oxygen or amide nitrogen of S240, backbone carbonyl oxygen or amide nitrogen of P241 and the side-chain amine nitrogen of K256. Heatmaps of lipid headgroup interactions were generated by tracking the position of the side-chain amine nitrogen of K256, K271, K275 and K260 and the position of phosphatidylserine phosphate oxygens and carboxylic oxygens, phosphatidylethanolamine phosphate oxygens, phosphatidylcholine phosphate oxygens, phosphatidylinositol phosphate oxygens, phosphatidic acid phosphate oxygen and ceramide glycerol hydroxyl oxygens. The same method was used for obtaining the average position of the backbone of the δC1b domain using the Cα carbons. Occupancy was defined as the distance between the atoms listed above being within ≤6 Å. Insertion depth was determined as the z-position of Gly253 relative to the average z-position of the PS phosphate groups. The stability and equilibration of the system was also assessed by assessing insertion depth over time (Figure S9c). Tilt angle was determined by calculating a centroid for the upper third of the C1b domain (using residues M239, P241, G253, and V255) and the bottom third of the C1b domain (using residues P233, H246, D263, and V276). A vector passing through these two centroids forms an angle with a line perpendicular to the membrane normal. This angle was used to report the tilt of the C1b domain in the membrane. PyMOL [93] was used to observe the structure and trajectories for each system. Graphical plots of interaction data were generated in GraphPad Prism 9.

## 5. Conclusions

Through molecular dynamics simulations, we show that cholesterol interacts with the δC1b domain. Through topological changes and variations in δC1b-lipid interactions, the δC1b domain displays a significant decrease in cholesterol interactions when bound to bryostatin due to a shallow membrane insertion depth. This decrease in the δC1b domain’s interactions with cholesterol when bound to bryostatin could affect its likelihood of translocating to cholesterol-rich regions of the cell membrane, such as lipid rafts, which could alter substrate accessibility and degradation rates of PKC-δ, thereby influencing cell signaling. This work highlights the importance of including physiologically relevant lipids into simulations of membrane–protein interactions.

## Figures and Tables

**Table 1:** Membrane composition for δC1b-phorbol simulations.

Lipid	Upper Leaflet	Lower Leaflet
Cholesterol	14 (21%)	13 (22%)
POPC	13 (20%)	12 (20%)
POPE	18 (27%)	17 (29%)
SOPS	8 (12%)	7 (12%)
POPA	2 (3%)	1 (2%)
PLPIP35	4 (6%)	3 (5%)
CER160	7 (11%)	6 (10%)
Total	66	59

**Table 2:** Membrane composition for δC1b-bryostatin and δC1b-Merle27 simulations.

Lipid	Upper Leaflet	Lower Leaflet
Cholesterol	16 (22%)	16 (24%)
POPC	15 (20%)	13 (19%)
POPE	19 (26%)	18 (27%)
SOPS	8 (11%)	8 (12%)
POPA	3 (4%)	2 (3%)
PLPIP35	5 (7%)	4 (6%)
CER160	7 (10%)	6 (9%)
Total	73	67

## Data Availability

The data presented in this study are available on request from the corresponding authors.

## Supplementary Materials

The following supporting information can be downloaded at: https://www.mdpi.com/article/10.3390/ijms24054598/s1.

## Abbreviations

Protein Kinase C (PKC), Molecular Dynamics (MD), phosphotidylethanolamine (PE), phosphatidic acid (PA), phosphatidylinositol phosphate (PIP), ceramide (CER), phosphotidylserine (PS), phosphotidylcholine (PC), diacylglycerol (DAG), nuclear magnetic resonance (NMR).
