# Supplementary material for: Insertion Depth Modulates Protein Kinase C-δ-C1b Domain Interactions with Membrane Cholesterol as Revealed by MD Simulations
Source: Int J Mol Sci. 2023 Feb 27;24(5):4598. doi: 10.3390/ijms24054598 (PMC10002858; doi:10.3390/ijms24054598)
Supplement: Supplementary file 1 [file ijms-24-04598-s001.zip › ijms-2187658-supplementary.pdf]

# Supporting Information

# Insertion Depth Modulates Protein Kinase C-δ-C1b Domain Interactions with Membrane Cholesterol as Revealed by MD Simulations

**Patrick T. Judge [1], Sarah A. Overall [2,*] and Alexander B. Barnes [2,*]**

[1] Department of Biochemistry, Biophysics & Structural Biology, Washington University in St. Louis, St. Louis, MO 63130, USA
[2] Laboratory of Physical Chemistry, ETH Zürich, Zurich, 8093, Switzerland
* Correspondence: sarah.overall@phys.chem.ethz.ch (S.A.O.); alexander.barnes@phys.chem.ethz.ch (A.B.B.)

**Table of Contents**

**Figure S1:** ***Chemical structures of ligands used in this study***
**Figure S2:** ***Heatmaps of PS interactions with δC1b-phorbol and δC1b-bryostatin and δC1b-Merle27***
**Figure S3:** ***Interaction lifetimes of $PIP_3$ with δC1b***
**Figure S4:** ***Heatmaps of cholesterol interactions with δC1b-phorbol and δC1b-bryostatin and δC1b-Merle27***
**Figure S5:** ***Variation in the insertion depth of bryostatin correlates with cholesterol occupancy***
**Figure S6:** ***Insertion of cholesterol interacting residues***
**Figure S7:** ***Structural access of δC1b-Merle27 to cholesterol***
**Figure S8:** ***Dynamics of W252 when PKC-δ is bound to different ligands***
**Figure S9:** ***System minimization and equilibration analysis***

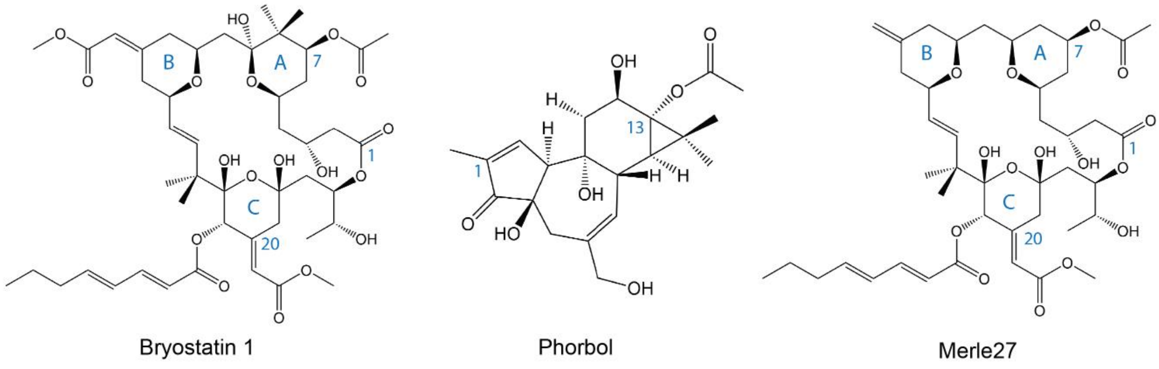


***Figure S1. Chemical structures of ligands used in this study.*** *Chemical structures of bryostatin 1, phorbol mono acetate and Merle17 used in the simulations presented.*

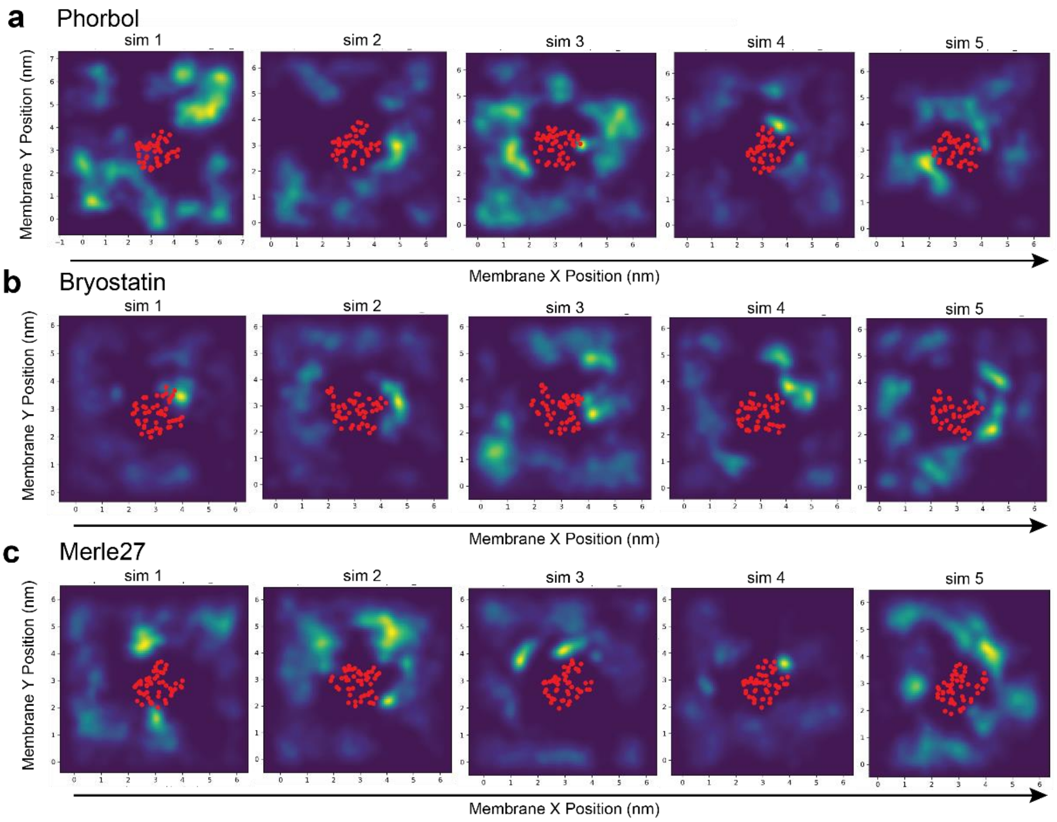


***Figure S2. Heatmaps of PS interactions with δC1b-phorbol and δC1b-bryostatin and δC1b-Merle27.*** *Heatmaps of the localization of PS in the inner leaflet of heterogenous membranes. All five simulations are shown for phorbol (a), bryostatin (b) and Merle27 (c). Lighter colors represent higher frequency of PS in that position. The red dots indicate the average position of the backbone Cα of δC1b. Each simulation was independently run for 500 ns.*

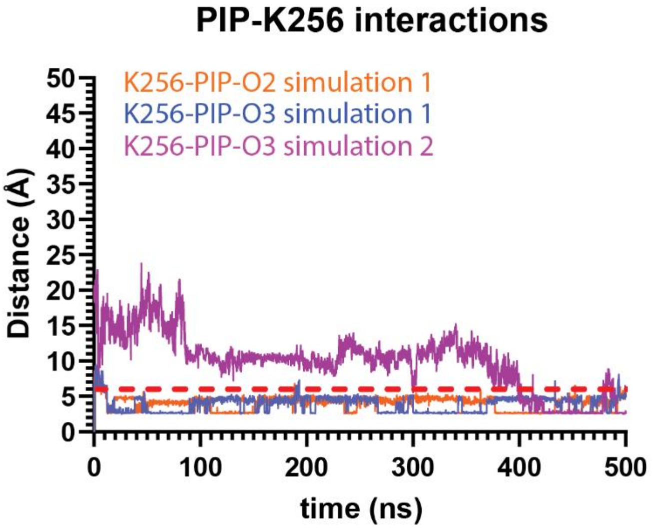


(**a**)

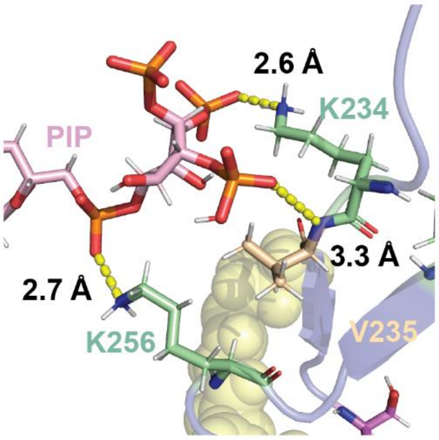


(**b**)

***Figure S3. Interaction lifetimes of PIP with δC1b.*** *a) Example interactions between phosphatidylinositol and K256 (interactions shown are extracted from bryostatin simulations but is representative of all simulations). The variation in interaction lifetime depending on PIP engagement is shown (simulation 1 vs simulation 2). It can also be seen that oxygen atoms from two different phosphates on a single PIP exchange with one another for the same K256 interaction (orange vs blue lines). b) Example of the structural interactions with K256 and neighboring K234 in bryostatin simulations plotted in a).*

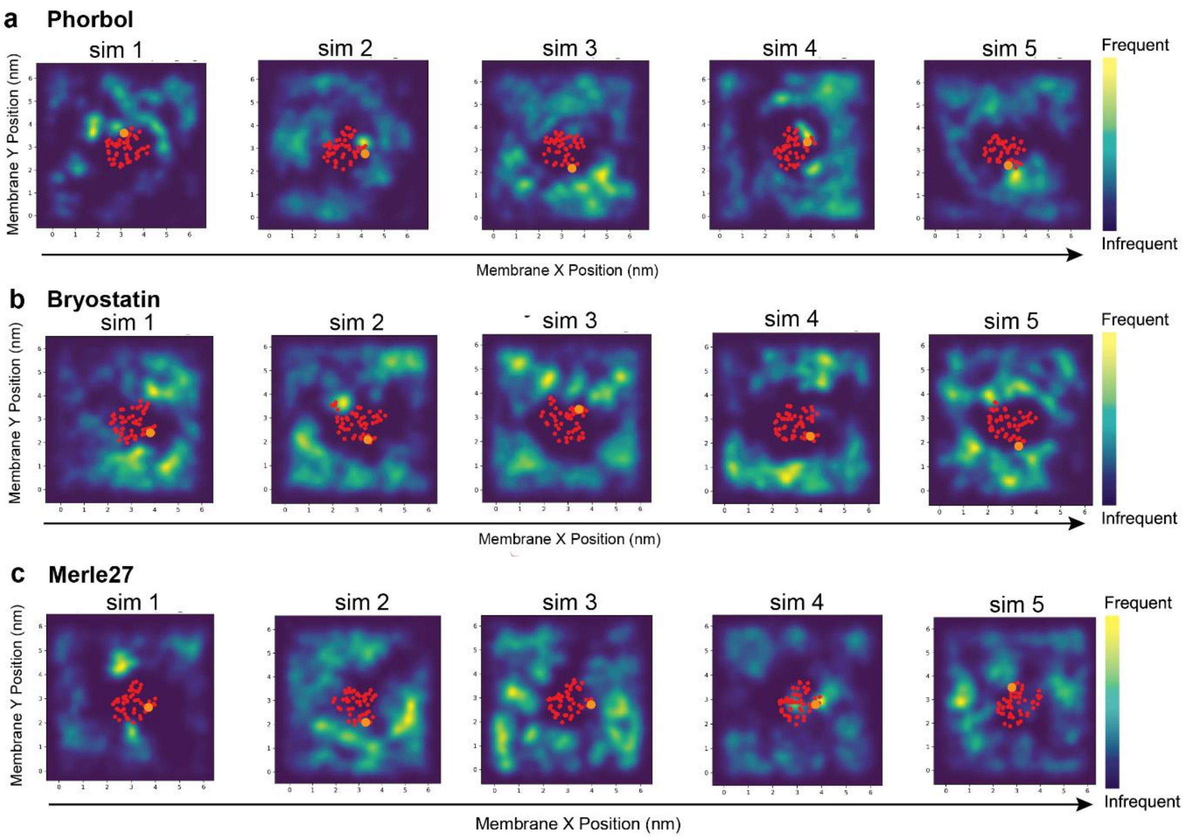


***Figure S4. Heatmaps of cholesterol interactions with δC1b-phorbol and δC1b-bryostatin and δC1b-Merle27.*** *Heatmaps of the localization of cholesterol in the inner leaflet of heterogenous membranes. All five simulations are shown. Lighter colors represent higher frequency of cholesterol in that position. The red dots indicate the average position of the backbone Cα of δC1b. The orange dot indicates the position of L250. Each simulation was independently run for 500 ns.*

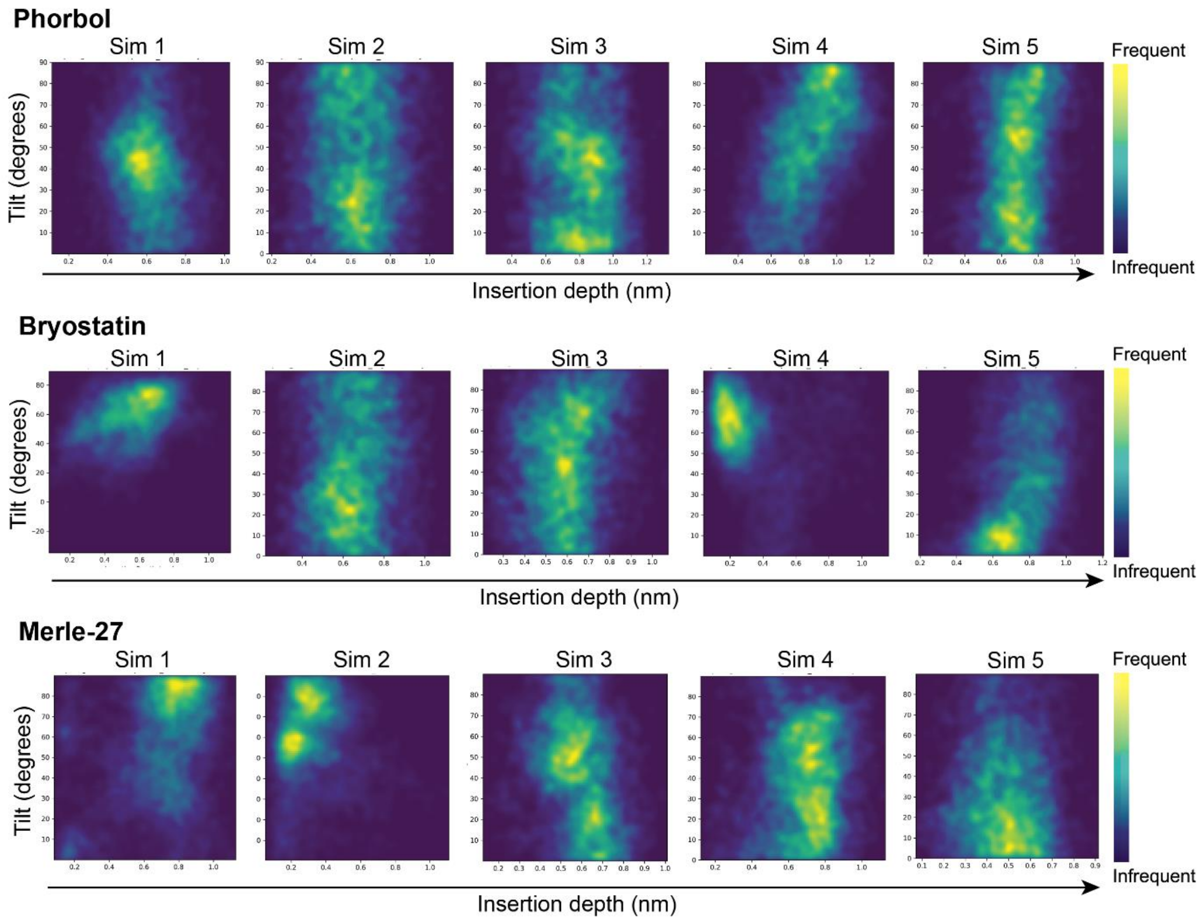


***Figure S5. Variation in the insertion depth across simulations.*** *Heatmaps of the tilt angle and insertion depth of phorbol simulations (top panels), bryostatin (middle panels) and Merle-27 (bottom panels) are shown across all 5 simulations performed per drug molecule. Variation in the insertion depth of bryostatin correlates with cholesterol occupancy**.** Heatmaps of δC1b-bryostatin topology across the 5 simulations. Sim5 shows deeper insertion than the other simulations and is the simulation in which cholesterol interactions with the δC1b-bryostatin complex were observed.*

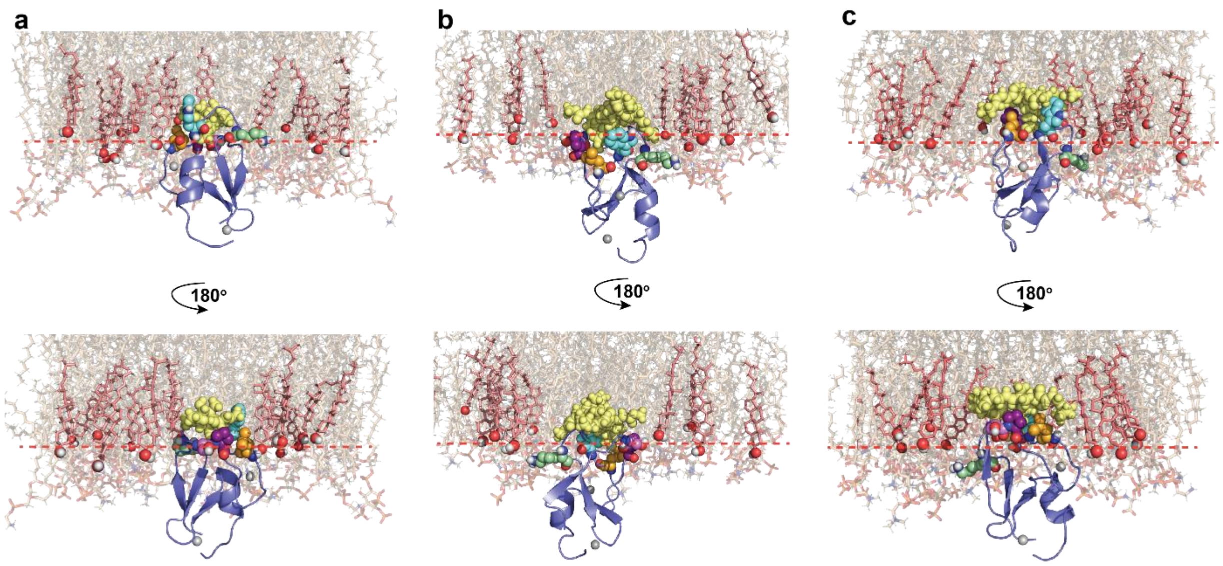


***Figure S6. Insertion of cholesterol interacting residues.*** *Structural snapshots of δC1b insertion when complexed to phorbol (a), bryostatin (b) and Merle27 (c). Ligands are represented as yellow spheres, L250 (orange), W252 (cyan), K256 (pale-green), S240 (pink) and P241 (purple) are shown in sphere representation. Cholesterol molecules are shown as red sticks with the hydroxyl group highlighted as a red sphere. The interfacial region is indicated by the dashed red line.*

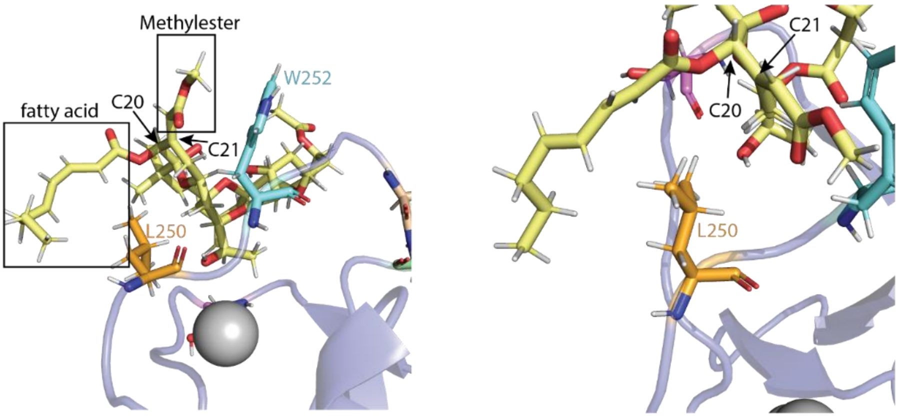


***Figure S7. Structural access of δC1b-Merle27 to cholesterol.*** *Structural snapshots showing the position of the Merle27 C20 fatty acid chain and the C21 methyl ester (black boxes) relative to L250 and W252. Clear steric hinderance about this residue is observed.*

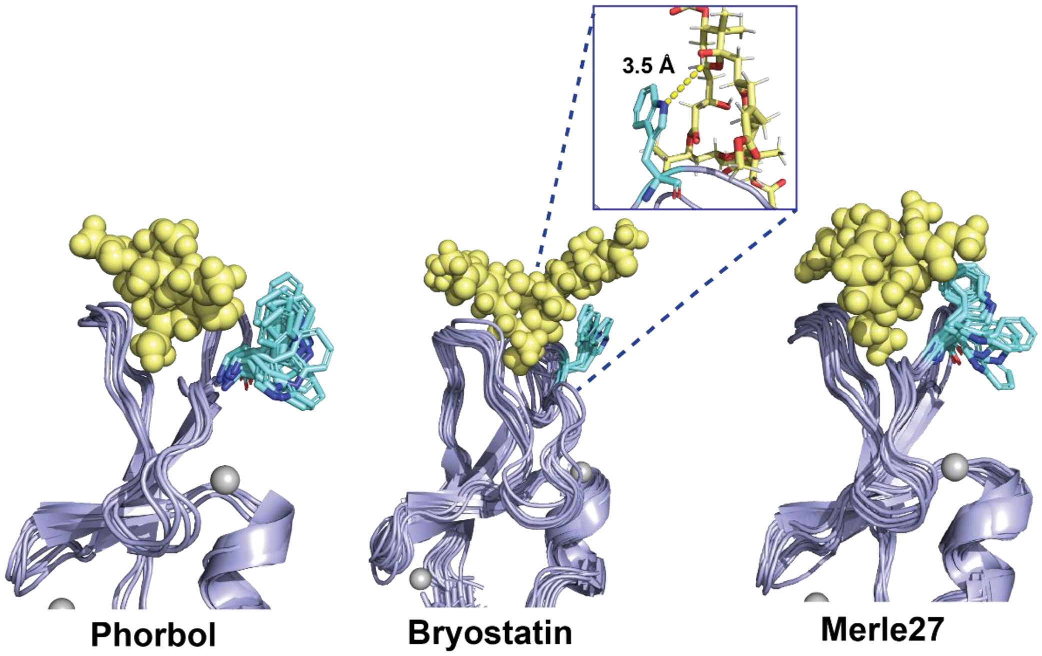


***Figure S8. Dynamics of W252 when PKC-δ is bound to different ligands***. *Overlays of each frame of a 500 ns simulation are shown and are representative of 5 simulations. W252 is shown in cyan and the indole and backbone nitrogen shown in blue. Ligands are shown as yellow spheres. The full range of W252 side chain positions in each simulation are shown. The inset highlights the interaction between bryostatin and the W252 indole nitrogen.*

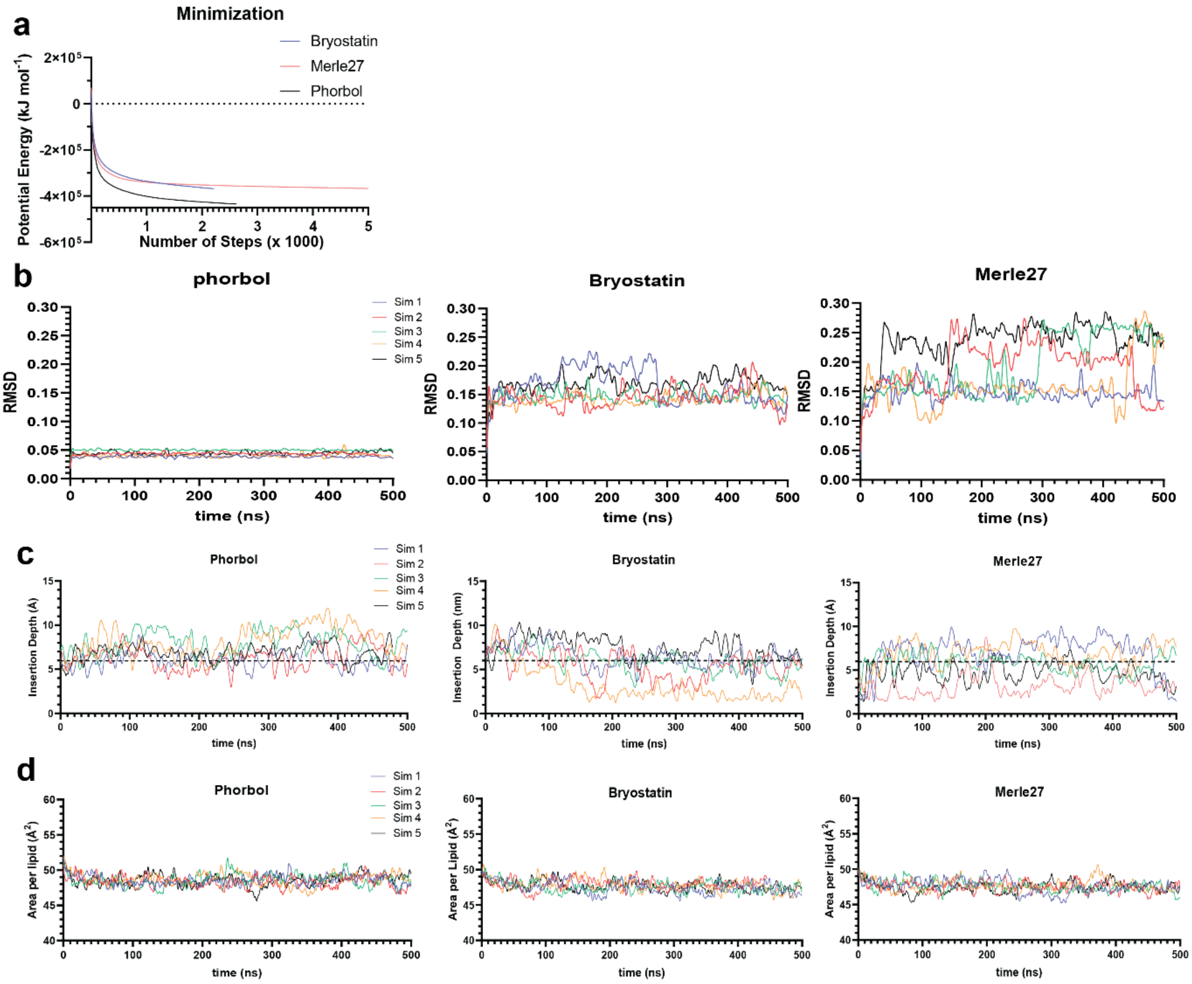


***Figure S9. System minimization and equilibration analysis.*** *a) System minimization using steepest descent method. Bryostatin and phorbol systems converged to a* $F_{max}$ *of <1000 kJ/mol/nm within 5000 steps. Merle systems converged to an* $F_{max}$ *of 6329 kJ/mol/nm over 5000 steps. b) RMSD of ligand structures relative to δC1b receptor complex. RMSD values of the ligand over time. The data shows all 5 simulations from each drug-δC1B complex. c) Insertion depth of ligand-δC1b complexes as a function of time over each production run. The dashed line marks 6 Å to aid visual comparison of insertion depth between ligand complexes. d) Area per lipid plotted as a function of time over each production run indicating system equilibration and relative stability over the course of the simulation.*
